# On the applicability of x-ray strain imaging using the edge illumination technique in biomedical applications

**DOI:** 10.1088/1361-6463/adf452

**Published:** 2025-08-04

**Authors:** Carlo Peiffer, A Astolfo, M Endrizzi, C K Hagen, A Olivo, P R T Munro

**Affiliations:** Department of Medical Physics and Biomedical Engineering, University College London, Gower Street, WC1E 6BT London, United Kingdom

**Keywords:** x-ray phase contrast, edge illumination, strain imaging, soft tissue x-ray strain imaging, lab based x-ray phase contrast

## Abstract

Strain imaging using conventional x-ray tomography is a widely established technique for investigating the mechanical deformation of materials, including cement and batteries. However, its biomedical applications are primarily restricted to bone tissue due to the low contrast of soft tissues. X-ray phase contrast imaging, offering superior contrast-to-noise ratios in soft tissues, can in principle overcome this limitation. This study explores the feasibility of x-ray strain imaging for soft tissues using edge illumination (EI), a laboratory-based x-ray phase contrast technique. A phantom mimicking the mechanical properties of healthy and tumorous soft tissues, with a stiff inclusion invisible to conventional x-ray imaging, was tested alongside chicken soft tissue fixed in ethanol. While our study confirmed that EI phase contrast imaging provides improved contrast for such samples compared to absorption imaging, it also revealed a reduction in strain retrieval precision. Artefacts caused by absorbing bridges in the mask design and errors arising from differential phase signal integration, which vary spatially between scans, were identified as key limiting factors. Consequently, EI phase contrast strain imaging was unable to locate phantom inclusions based on mechanical contrast. However, EI’s capability to increase spatial sampling frequency without compromising the field of view improved strain retrieval precision using its absorption contrast beyond that achieved with conventional x-ray strain imaging. These findings highlight the potential and challenges of applying EI to strain analysis in soft tissues, providing insights into its limitations and opportunities for further improvement.

## 1. Introduction

The mechanical properties of biological tissues are known to be modified by disease [1]. For example, breast tumours [2], liver tumours and fibrosis [3, 4] and prostate tumours [5], are generally stiffer than their surrounding healthy tissue. This is why manual palpation has been used for centuries [6], if not longer, to diagnose disease. For this reason, so-called elastography approaches have been developed, which exploit a primary imaging modality to acquire three-dimensional images of tissue stiffness. Elastography images are acquired by tracking deformation caused by a mechanical loading mechanism which is either quasistatic or dynamic. The choice of loading mechanism is limited by the employed imaging modality. Dynamic loading, for example, is compatible with ultrasound [7], MRI [8], and optical coherence tomography [9]. In this approach, a harmonic or transient mechanical pressure wave is applied to the sample, and its propagation is imaged in real time. The local dynamic response can then be used to determine material properties such as the Young’s modulus. Quasistatic loading, on the other hand, does not require fast imaging and is therefore compatible with all imaging modalities. A sample is first imaged in an unloaded state. The sample is then loaded quasistatically, which means that the load is applied slowly enough for the sample to reach equilibrium before it is imaged a second time. The two images are then used to retrieve a strain field. The strain *ϵ* measures the relative deformation of a sample and in 1D the axial strain is defined as

\begin{align*} \varepsilon = \frac{\partial u}{\partial x}\end{align*}

where *x* is the space coordinate and *u* is the displacement in *x*-direction. For negative *ϵ*, the strain is compressive, while for positive *ϵ*, the strain is tensile. Under the simplest assumption that the load imparts uniform axial stress throughout the sample, the Young’s modulus *E* can be calculated by

\begin{align*} E = \frac{\sigma}{\varepsilon},\end{align*}

where *σ* is the axial stress and *ϵ* is the axial strain. Hence, *E* is inversely proportional to *ϵ* and therefore the axial strain distribution can be used as a proxy for the elasticity of the material. Different combinations of imaging modalities and loading mechanisms have been applied to various tissue types. For example, dynamic magnetic resonance elastography has been used to assess liver fibrosis [4], while ultrasound elastography, both static and dynamic, has been employed to investigate the liver, breast, thyroid, prostate, kidney, and lymph nodes [10]. Additionally, static optical coherence elastography has been utilised for margin detection in excised breast tumour tissue [11]. These elastography techniques have spatial resolution, contrast and penetration depth of the primary imaging modality matched to the target application. MRI, for example, has a penetration depth on the order of decimeters, but a comparatively low spatial resolution of 0.1–1 mm. Optical coherence tomography, on the other hand, has a resolution on the order of micrometres [12], but its penetration depth is limited to a few millimetres at most [13]. X-ray imaging, however, has the advantage of a penetration depth on the decimeters scale and resolution of tens of microns. X-ray imaging is thus superior to other imaging modalities in terms of being able to simultaneously acquire images with both high spatial resolution and high penetration depth. So far x-ray strain imaging has been predominantly applied to industrial applications with high density materials having strong absorption contrast [14, 15], and on select biological tissues such as bone [16–18]. Usually, a series of quasistatic CT scans, each corresponding to different mechanical loads is recorded and then a digital volume correlation (DVC) algorithm [16] is employed to retrieve a deformation or strain field. The strain precision and spatial resolution of these algorithms depend on the visible characteristic feature sizes of the sample and the imaging modality’s properties such as contrast-to-noise ratio (CNR) and spatial resolution. The CNR is an image quality metric that quantifies how well image details can be distinguished from the background in the presence of noise. It is defined as

\begin{align*} \mathrm{ CNR} = \frac{|S_\mathrm{ d}-S_\mathrm{ b}|}{S_\mathrm{ N}},\end{align*}

where $S_\mathrm{ d}$ is the mean intensity of an image detail, $S_\mathrm{ b}$ is the mean signal of a surrounding background region and $S_\mathrm{ N}$ is the noise strength obtained by calculating the standard deviation of the background signal. For soft tissues, the CNR is usually low in absorption images, hence attempts have been made to exploit phase contrast in CT images of intervertebral disk tissue using Synchrotron radiation [19, 20]. Synchrotrons, however, are scarce and therefore unfeasible for widespread clinical applications.

In this study we investigate the possible use of lab-based x-ray strain imaging using the edge illumination (EI) technique [21] and a DVC approach to retrieve the strain field. We investigate a tissue mimicking phantom with a stiff inclusion (that could resemble a tumour in breast tissue), which is invisible to all contrast mechanisms of EI. We exploit the strain contrast to identify the inclusion and validate our findings with a finite element analysis (FEA). We furthermore compare the performance of DVC on the different accessible image contrast mechanisms of EI and investigate EI’s ability to increase the resolution while maintaining the field of view.

## 2. Methods

### 2.1. Phantom design

The phantoms were designed to mimic the mechanical and x-ray imaging properties of a breast tumour in healthy tissue. Literature values of Young’s modulus for healthy and tumorous tissue range from 3 kPa for normal fat tissue and normal fibroglandular tissue to 20 kPa for intermediate-grade and 42 kPa for high-grade infiltrating ductal carcinoma [2]. Gel wax has been previously demonstrated as a good multimodal phantom material as it allows a range of physical properties to be controlled [22]. Of particular interest to this study, its stiffness can be increased by the addition of paraffin wax. Furthermore, an x-ray absorption image does not provide sufficient contrast to allow for delineation of volumes containing higher concentrations of paraffin wax, meaning that volumes of stiffer material are indistinguishable from softer regions using only x-ray absorption imaging. This simulates the frequently encountered scenario where tumour tissue is hard to distinguish from healthy tissue in a conventional x-ray CT.

However, we hypothesise that this lack of direct tissue discrimination can be overcome by quasistatic x-ray strain imaging. In this approach, the strain retrieval is based on the application of DVC and therefore on the tracking of the movement of small features during compression. In DVC, volume subsets of a reference image are correlated with subsets of a deformed image. The correlation works better the more distinct and finer the subvolumes’ texture is Disney *et al* [19, 20] showed that using Synchroton x-ray phase contrast reveals this fine texture also in soft tissue (in this case cartilage) and therefore DVC could be applied successfully to soft tissue. This has not been shown with laboratory x-ray sources yet. Previously, using a lab-based x-ray source, calcium carbonate particles were used in a phantom in order to create the typical features that are visible in an x-ray image of a human breast [23] but the particles had an inherently strong absorption contrast and they aggregated around the inclusion, enabling the inclusion to be identifiable without the help of the strain image. Instead, we wanted to make a phantom where the stiff inclusion is radiographically indistinguishable from the surrounding matrix, with a texture that is enhanced when using phase contrast, as is the case when imaging biological tissue. We used PMMA microspheres (Product 178 760 250, Poly(methyl methacrylate) beads, $\unicode{x2300} = 50$ to $150\,\mu$m, Fisher Scientific) as a source of contrast in gel wax to create a more realistic phantom, both in terms of its mechanical properties and x-ray contrast. In addition to the atomic composition of a material, its density is a key factor in determining the material’s x-ray absorption coefficient. Because PMMA’s density (1.18 g cm^−3^) is much closer to that of gel wax (0.9 g cm^−3^) compared with that of ${\mathrm{CaCO}_3}$ (2.5 g cm^−3^), its x-ray absorption coefficient should be closer to gel wax, leading to low absorption contrast between the two materials. This is a favourable property because, apart from calcifications, most of the expected features in soft tissues like breast tissue have low absorption contrast. Another advantage of the more similar density is that the PMMA particles can be distributed more homogeneously during the gel wax hardening phase, while ${\mathrm{CaCO}_3}$ particles tend to drop quickly due to gravity.

**Figure 1.**
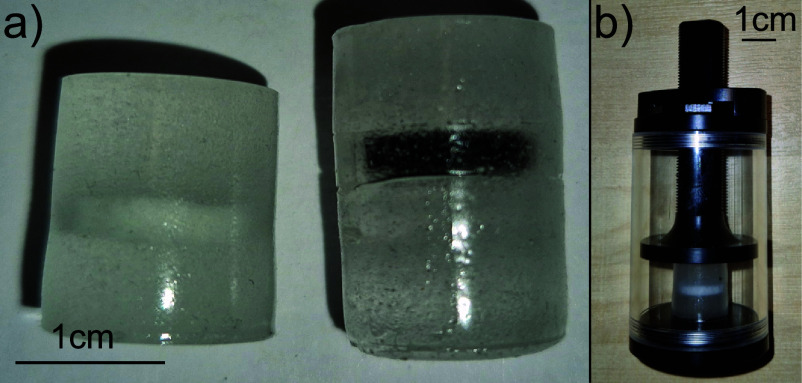
(a) Photograph of phantom A on the left and phantom B on the right. ${\mathrm{TiO}_2}$ powder was incorporated into the inclusion of phantom A whereas the inclusion of the phantom B was dyed black with ink. (b) Photograph of the custom made compression stage. A displacement is prescribed by turning a hand screw.

The two phantoms created as part of this study are shown in figure 1(a) and were produced as follows: first, the inclusion was prepared by melting and mixing the required materials, as detailed below. Because PMMA has a melting point of 105${^\circ}$, gel wax was heated up to only 100${^\circ}$, which was enough to melt the gel wax but not PMMA. Paraffin wax was then added such that it made up 10$\%$ of the mixture’s mass. Then, using a mechanical stirrer, 15 w$\%$ PMMA microspheres were homogeneously distributed into the melt. Since the inclusion is indistinguishable from the surrounding matrix using x-ray imaging, for one of the phantoms (phantom A) we added 1 w$\%$ ${\mathrm{TiO}_2}$ microparticles (Product 224 227, Titanium(IV) oxide particles, $\unicode{x2300} < 5\,\mu$m, Sigma Aldrich) to the inclusion. This creates absorption and scattering contrast between the inclusion and the matrix, making it possible to visually validate the retrieved strain field in the CT reconstruction. For visual verification of the shape and position of the inclusion in the optical regime only, a second phantom (phantom B) was created by mixing a few drops of black ink (Black Indian Ink, Graff-City) into the inclusion instead of ${\mathrm{TiO}_2}$ microparticles. The ink, unlike the microparticles, was not visible in the CT reconstruction. The inclusions were cast into cylindrical metal moulds with a 10 mm diameter. After solidifying by cooling to room temperature, the material was cut with a scalpel into cylindrical blocks of around 3 mm height. In order to create the matrix, gel wax was melted and then 15 w$\%$ PMMA microspheres were stirred into it. We measured the Young’s modulus for the matrix and inclusion components of the phantom quasistatically using an electromechanical universal testing machine (Instron 5944). We ensured that the sample was in full contact with the loading pistons, then the sample was axially compressed by 0.1 mm within 10 s and this displacement was held for 310 s for the material to relax. Force measurements were taken at the end of the relaxation period. Using 2 we found that introducing PMMA microspheres into gel wax actually reduced the Young’s modulus of plain gel wax from 16.1 ± 1.3 kPa (compared to 17.4 ± 1.4 kPa [22] found in the literature) to 3.2 ± 1.0 kPa. The inclusion’s Young’s modulus was 13.9 ± 1.3 kPa.

### 2.2. EI

**Figure 2.**
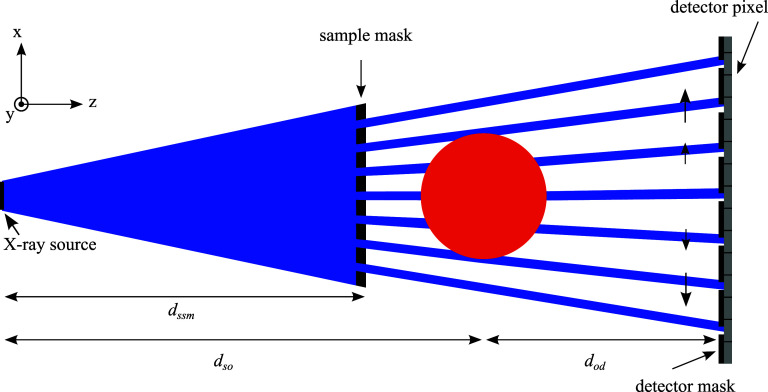
Sketch of an edge illumination system (not to scale). The x-ray cone beam is shaped into independent beamlets by means of a sample mask. A detector mask is introduced immediately before the detector so that any deviation of a beamlet is transformed into a change in detected intensity.

**Figure 3.**
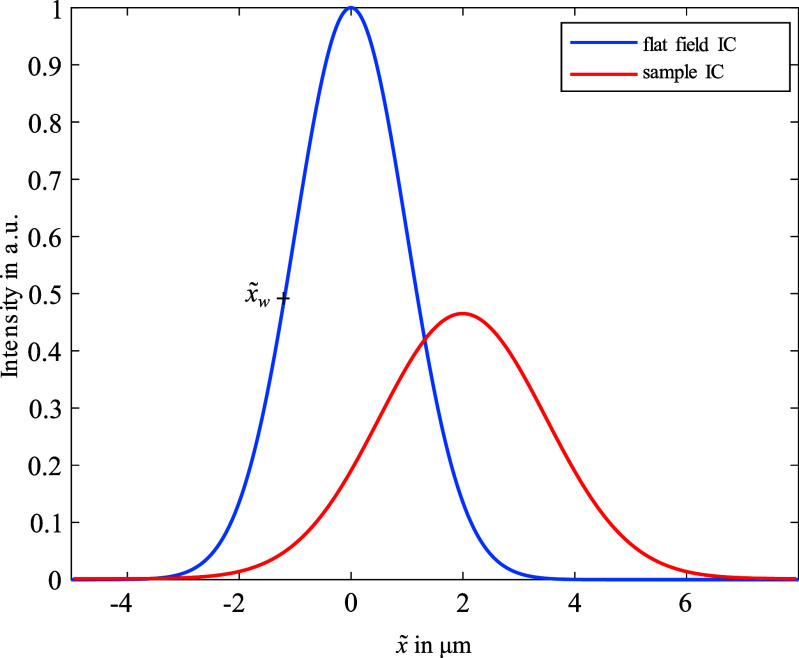
Example plots of a flat field IC in blue and an IC after introduction of a sample in red. Absorption leads to a reduction of the area under the curve, refraction leads to a shift of the curve and subresolution scattering leads to a broadening of the curve. $\tilde{x}$ denotes the lateral shift between the sample and the detector mask. $\tilde{x}_w$ shows a commonly chosen working point for single-shot scans.

Figure 2 shows a sketch (not to scale) of the EI setup that was used for this study. A Rigaku MicroMax 007HF x-ray source with a Molybdenum target was operated at 40 kvp and 30 mA with a focal spot FWHM of 70 *µ*m. The x-ray cone beam was shaped into x-ray beamlets by the so-called sample mask (pitch = 79 *µ*m, aperture = 10 *µ*m); these beamlets were then analysed by the detector mask (pitch = 98 *µ*m; aperture = 17 *µ*m), positioned as close as possible to the detector (Hamamatsu CMOS Flat Panel Sensor (C9732DK)), which was placed 86 cm downstream of the x-ray source. In this setup we used a column skipping detector mask, which means that every second pixel column of the detector is fully covered in order to reduce pixel cross-talk. When the sample mask is scanned in x-direction, the intensity profile recorded by a pixel as a function of sample mask displacement is called illumination curve (IC). The IC can be calculated by convolving the beamlet profile with the shape of the transmitting region of a slit of the detector mask. Figure 3 shows an example flat field IC with no sample in the beam where $\tilde{x}$ describes the lateral shift between the two masks. Introducing a sample immediately behind the sample mask changes the beamlet’s intensity profile, which we will call sample IC. Absorption leads to a reduction of the area under the curve, refraction leads to a translation of the curve and sub-resolution scattering leads to a broadening of the curve. The effect of these contrast mechanisms can be separated by the phase retrieval algorithms described later. Another consequence of using a mask to structure the beam is that, for EI, the resolution is determined by the mask aperture size rather than the pixel size. With a source to sample mask distance ($d_\mathrm{ssm}$) of 68 cm and a source to sample distance ($d_\mathrm{so}$) of 72.5 cm the magnification of the sample mask at the sample is $M = \frac{d_\mathrm{so}}{d_\mathrm{ssm}} = 1.07$. The resolution is therefore determined by the magnified sample mask aperture of 10.7 *µ*m and the sampling frequency is limited by the magnified sample mask pitch at the sample which is 84.2 *µ*m. In order to increase the sampling frequency, dithering is performed by scanning the sample in sub-mask-pitch steps through the beamlets and the acquired frames are stitched together in order to achieve a projection with an increased sampling frequency, while maintaining a field of view that is large (∼3 × 3 cm) compared to conventional microfocus CT, where high resolution is achieved using geometric magnification only.

### 2.3. Retrieval methods

#### 2.3.1. Multi IC point retrieval.

In order to retrieve the absorption, refraction and scattering signals, frames on multiple points of the IC were acquired. We followed the procedure proposed by Maughan Jones *et al* [24], which has shown good results even when the IC has an intensity offset due to partial transmission, in which each pixel the ICs of the pixel and its two neighbours (in the direction of phase sensitivity) were fitted to the sum of three displaced Gaussians, represented mathematically as

\begin{align*} I\left(x\right) = \sum_{n = -1}^{1} t_n \frac{\exp\left(\left(-x-r_n+np\right)^2/2c_n^2\right)}{\sqrt{2\pi c_n^2}}\end{align*}

where *p* is the period of the sample mask and *t_n_*, *r_n_*, *c_n_* are the fitting parameters. Doing this for the flat field IC and sample IC and using only the parameters of the central Gaussian enables to obtain:

\begin{align*} a &amp; = -\mathrm{ln}\left(t_\mathrm{sample}/t_\mathrm{flat}\right) \nonumber\\ \phi\left(x\right) &amp; = \int_0^x \frac{2\pi}{\lambda}\left(r_\mathrm{sample}-r_\mathrm{flat}\right)/\mathrm{d}_\mathrm{od} \mathrm{d} x^{^{\prime}} \qquad s^2 = c_\mathrm{sample}^2-c_\mathrm{flat}^2\end{align*}

where *a* is the absorption signal, *φ* is the phase, *λ* is the effective wavelength, $d_\mathrm{od}$ is the sample to detector distance, *x* is the coordinate in the direction of phase sensitivity and *s*^2^ is the scattering signal. The signals *a*, *φ* and *s*^2^ all increase linearly with thickness, hence they are amenable to imaging by CT. To account for changing illumination conditions, such as mask or source drifts, the refraction signal for each dithering step was normalised by subtracting the average intensity of a background region of interest (ROI) before the integration step was performed.

#### 2.3.2. Single-shot retrieval.

The acquisition time for a fully dithered (up to the Nyquist limit) and multi IC point CT data set exceeds one day. In order to speed up the scans and save x-ray dose, the spatial sampling frequency can be decreased, depending on the characteristic feature sizes, while maintaining a sufficient image quality. Furthermore, it is possible to use only a single frame per dithering step to obtain a phase enhanced projection by choosing the point of steepest slope of the flat field IC as the working point $\tilde{x}_w$ (see figure 3). At this point on the IC, the resulting change in recorded intensity in response to a shift of the IC is maximised. Making the approximation that the ratio $\gamma = \delta/\mu$, where *δ* is the real decrement of the x-ray refractive index $n = 1-\delta + \mathrm{i}\beta$ and *µ* is the x-ray absorption coefficient, stays constant throughout the sample [25] and assuming the beamlet shift due to refraction to be small compared with the width of the IC, the equation

\begin{align*} &amp; \int \mathrm{d}z\mu_\mathrm{eff}\left(x,y,z\right)\nonumber\\ &amp;\, = -\mathrm{ln} \left[ \mathscr{F}^-1\left( \frac{\mathscr{F}\left(I/\mathrm{IC}\left(x_w\right)\right)}{1-\mathrm{i}2\pi \mathrm{IC}\left(x_w\right)/\frac{\partial}{\partial x}\mathrm{IC}\left(x_w\right)\gamma_\mathrm{eff}f_x+4\pi^2d_\mathrm{od}\gamma_\mathrm{eff}f_y^2}\right) \right]\end{align*}

can be obtained [26]. Here, *I* is the signal intensity, $f_x,f_y$ are the reciprocal space coordinates, *x_w_* is the IC working point and $\gamma_\mathrm{eff}$ is the effective proportionality value for the material of interest at the employed x-ray spectrum. $\mu_\mathrm{eff}(x,y,z)$ is therefore CT reconstructible. The approximation of $\gamma_\mathrm{eff}$ being constant throughout the sample is strictly valid only for a single material, but experimentally it has been shown that for chemically similar materials like different types of soft tissue this approach provides high image quality, although the retrieved images are not quantitative [27, 28]. The optimal $\gamma_\mathrm{eff}$ is found in an iterative process such that the profile across a material interface in the CT reconstruction is as similar as possible to a step function, which is assessed visually [29].

### 2.4. Compression test

CT data sets of a sample are acquired in preloaded and loaded states. Each projection is retrieved following one of the previously described approaches and then 3D volumetric images are reconstructed using the FDK cone beam reconstruction algorithm and a customisable vector geometry implemented in the ASTRA toolbox [30, 31]. DVC is conducted with the extension module ‘XDVC’ from the commercial software package AVIZO 3D 2022.1 [32]. First, the loaded CT volume is rigidly registered to the preloaded CT volume. The 6 translational and rotational degrees of freedom are optimised such that the sum of squared differences between corresponding voxels is minimised. This step is done to minimise any rigid motion that has happened between the two scans. The registered CT volume is then resampled onto the grid of the preloaded CT volume. Since AVIZO’s global DVC algorithm [33] is used, a homogeneous tetrahedral mesh that spans the entire sample is created. In this context, ‘homogeneous’ means that the mesh is optimized to ensure that cell sizes have the lowest possible variance. To achieve faster and more reliable convergence of the DVC algorithm, an initial coarse mesh is used and refined progressively in steps. The displacement field obtained at each step serves as the initialisation for the subsequent step. Generally, the precision in the strain retrieved by DVC decreases as the mesh becomes finer [34]. In a DVC compression test, it is therefore important to conduct a strain precision analysis in order to quantify the sensitivity of the test method and to ensure a mesh size with acceptable precision for the application is chosen.

### 2.5. DVC precision analysis

DVC strain precision can be estimated by performing strain retrieval on two repeated CT scans of the same sample in an unloaded state [16]. Since no deformation occurs between CT acquisitions, the strain should be zero for all the nodes of the mesh and therefore the standard deviation $\sigma(\varepsilon)$ of the retrieved strain field can be used as a measure of precision. We calculate standard deviation as

\begin{align*} \sigma\left(\varepsilon\right) = \sqrt{\frac{\sum_1^N \left(\varepsilon_i-\bar{\varepsilon}\right)^2}{N-1}},\end{align*}

where *N* is the number of sampled values of strain and $\bar{\varepsilon}$ is the mean value of all the sampled values of strain. In a strain precision analysis, *σ* is calculated and plotted for several mesh sizes in order to determine the best trade-off between strain precision and spatial resolution for the specific application.

### 2.6. FEA

In order to validate the retrieved strain field obtained using DVC, a FEA was conducted using the commercial software Ansys^®^ Academic Research Mechanical, Release 19.0. A pair of 3D images of phantom A were used to construct the model. The matrix and the inclusion were segmented using thresholding and converted into finite element meshes. The experimentally determined Young’s moduli (see Phantom design) were assigned to the respective materials. The contact between the compression rig and the phantom was chosen to be a ‘sliding contact’ under the influence of friction. Because the coefficient of friction between gel wax and polycarbonate (material of the loading pistons) was unknown, a range of values between 0.3 and 0.8 were trialled; however, the result remained qualitatively the same. For this paper, a coefficient of friction of 0.6 was chosen. One of the loading pistons was kept static while a compressive displacement of 0.5 mm (as was in the physical experiment) was applied to the other one. The contact mechanism between the matrix and the inclusion was set to ‘bonded’. Two finite element models were then solved: one where the phantom consisted of the matrix material only and another one consisting of a matrix containing an inclusion.

## 3. Results

All CT reconstruction data supporting this study are available in [35].

### 3.1. Identification of inclusion by strain field

**Figure 4.**
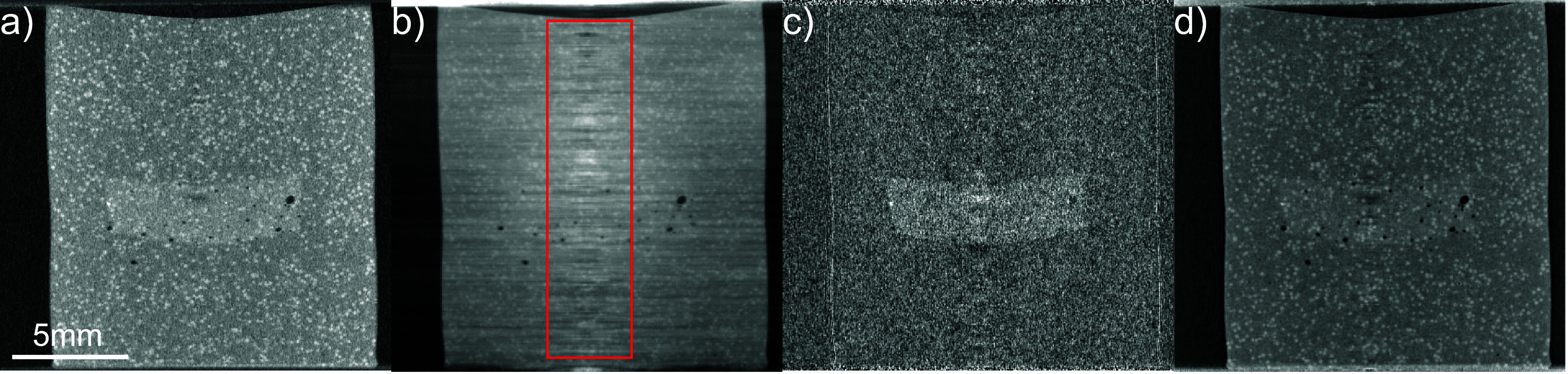
Longitudinal CT slices through phantom A for the different available image contrast mechanisms using two dithering steps and five IC points. (a) Retrieved absorption. (b) Retrieved phase.(c) Retrieved scattering signal. (d) Single-shot retrieval. The stiff inclusion is easily visible in the absorption, scattering and single-shot CT reconstruction because of the addition of ${\mathrm{TiO}_2}$ powder. The PMMA microspheres show the highest visibility in the absorption and single-shot CT slice. The retrieved phase CT reconstruction shows strong horizontal streak artefacts that are likely caused by the combination of decreased beam intensity behind the masks’ bridges and noise accumulation in the integration of the refraction angle required to obtain the total phase shift. Furthermore, artefacts along the rotation axis, marked in red, are visible. These are ring artefacts, smeared by horizontal jittering, and are most pronounced in the retrieved phase reconstruction.

Figure 4 shows CT slices through phantom A for different contrast mechanisms and retrieval methods. Visually, the best reconstruction is achieved using the retrieved absorption projections (figure 4(a)). The PMMA microspheres are seen as bright dots and the inclusion is visible because of the ${\mathrm{TiO}_2}$ powder’s additional absorption. The reconstruction using phase (figure 4(b)) is corrupted by streak artefacts. These are likely caused by both the absorbing bridges interrupting the vertical slit-shaped apertures in the mask design and the integration of refraction angles needed to obtain the phase projections (equation (5)), however, this is still under investigation.

The scattering contrast CT slice (figure 4(c)) shows the inclusion very clearly because the ${\mathrm{TiO}_2}$ powder is highly scattering. Apart from this, very few other features are visible, which is expected because the refractive index is constant inside the PMMA microspheres ($\unicode{x2300} = 50 $ to $ 150\,\mu$m) and inside the gel wax on the scale of the aperture size and therefore no subresolution scattering should take place. The single-shot reconstruction (figure 4(d)) was optimised for the material interface between gel wax and PMMA. It closely resembles the retrieved absorption reconstruction but appears blurred, an inherent effect of the filter used for single-shot phase retrieval. In particular, the inclusion is more difficult to discern. However, the CNR between the PMMA microspheres and the gel wax matrix is higher than in the retrieved absorption reconstruction (5.6 vs 3.4).

Furthermore, in all four reconstructions, an artefact along the rotation axis (delineated in red in figure 4(b)) is visible, most prominently in the retrieved phase reconstruction. These are ring artefacts, which have previously been shown to be effectively suppressed by introducing small, random sample displacements during acquisition [36, 37], and are reduced here through the same method, which we refer to as jittering in the following. This technique involves moving the sample horizontally by random, yet known, multiples of the demagnified mask pitch for each rotation angle, which is accounted for in the ASTRA reconstruction geometry. For this, a NEWPORT motor (XML210-S) with a positioning accuracy of 1.5 *µ*m and bi-directional repeatability of 0.04 *µ*m was employed. Random integers between −4 and 4 were obtained by applying the ‘randrange’ function of the python module ‘random’.

The rationale behind this technique is that each projection is sampled by a different set of detector pixels, each with a slightly different response. Over the course of the full set of projections, detector variability can therefore be averaged out [37], leading to a considerable reduction in ring artefacts. The aim of this technique is to blur out ring artefacts in CT reconstructions caused by non-linear pixel responses. While this approach effectively reduces artefacts in the absorption reconstruction, the rings remain prominent in the retrieved phase reconstruction, as the phase integration step amplifies the influence of non-linear pixels.

One potential drawback of this method is a slight loss of image resolution, which depends on the positioning precision and accuracy of the translation stage used. In our case, however, this effect is negligible, as the positioning accuracy (1.5 *µ*m) and repeatability (0.04 *µ*m) of the employed motor are substantially smaller than the jittering steps.

**Figure 5.**
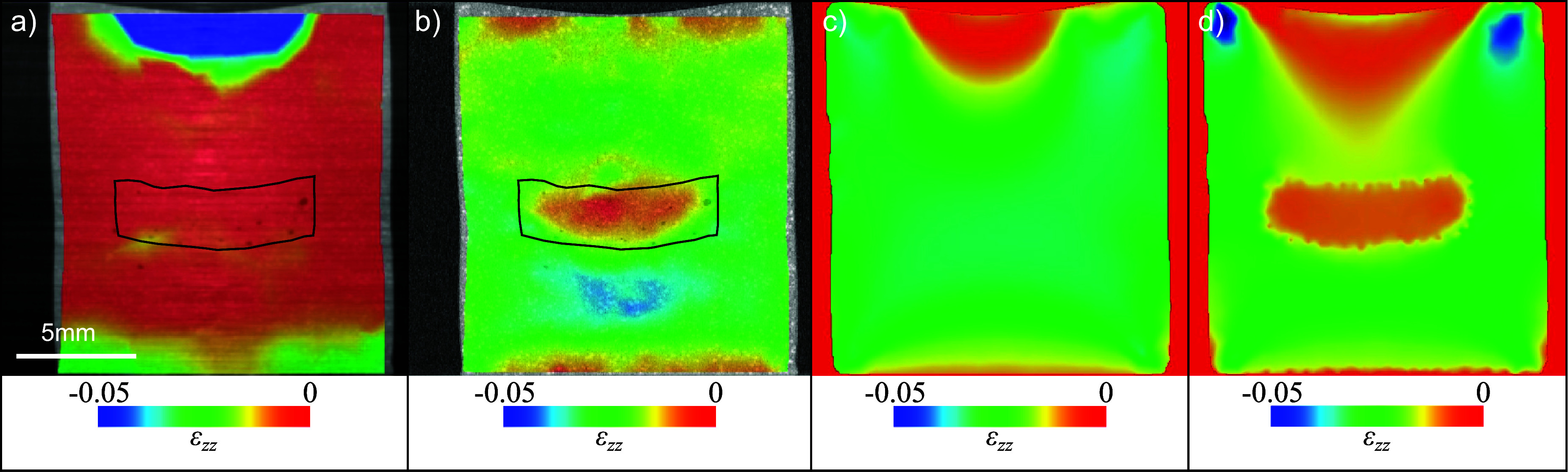
(a) Phase CT slice through phantom A overlaid with *ϵ*_*zz*_ strain field retrieved with DVC using a mesh size of 50 voxels. The inclusion is delineated in black. (b) Retrieved absorption CT slice through phantom A overlaid with *ϵ*_*zz*_ strain field retrieved with DVC using a mesh size of 20 voxels. A region of low strain coincides with the location of the stiff inclusion that is outlined in black. (c) *ϵ*_*zz*_ strain field obtained with FEA assuming the phantom to consist only of the matrix material without the inclusion. (d) *ϵ*_*zz*_ strain field obtained with FEA accounting for the stiffer inclusion.

In a compression experiment, phantom A was scanned in a preloaded state, where the loading pistons were adjusted using a hand screw until they just made contact with the phantom on both sides. A second scan was performed after the phantom was axially compressed by 0.50 ± 0.04 mm in the *z*-direction. Figure 5(a) shows a retrieved phase CT slice of the phantom in its preloaded state, overlaid with the corresponding distribution of the normal strain *ϵ*_*zz*_ in the *z*-direction, calculated via global DVC with a mean mesh size of 50 voxels (2.1 mm). This mesh size was chosen because with a smaller mesh the DVC algorithm did not converge anymore. Under compressive loading, a negative strain is expected, with reduced amplitude at the inclusion’s location. However, the retrieved strain displays an unphysical region of positive strain across most of the sample, contradicting the expected behaviour. This discrepancy arises due to the previously mentioned artefacts, which prevent accurate strain field determination.

Figure 5(b) presents a retrieved absorption CT slice of the phantom in its preloaded state, overlaid with the corresponding *ϵ*_*zz*_ strain field, obtained using global DVC with a finer mesh size of 20 voxels (0.84 mm), which was chosen because the DVC algorithm still converged at this resolution. The strain field highlights a region of low compressive strain within the inclusion location and an area of increased compressive strain between the inclusion and the phantom’s bottom boundary. These results suggest that the phantom is not in full contact with the top loading piston due to a meniscus formed during the gel wax cooling process. Consequently, the assumption of uniaxial loading may not hold, and the strain field cannot simply be approximated as the inverse of Young’s modulus. To validate the strain fields retrieved from phase and absorption CT to investigate the impact of boundary conditions or phantom shape, a FEA was performed. The FEA incorporated segmentation of the inclusion and matrix, along with the experimentally measured Young’s moduli.

Figure 5(c) shows the *ϵ*_*zz*_ strain field calculated by FEA under the assumption that the phantom consists solely of the matrix material, without an inclusion. The influence of the meniscus is evident in the low strain observed at the top of the phantom. In contrast, figure 5(d) presents *ϵ*_*zz*_ calculated by FEA using realistic Young’s moduli for both the matrix and the inclusion. While the meniscus effect remains visible as a low-strain region at the top, an additional near-zero strain region appears within the inclusion. As expected, the simulated strain field more closely matches that obtained from absorption CT reconstruction than the one from phase CT.

**Figure 6.**
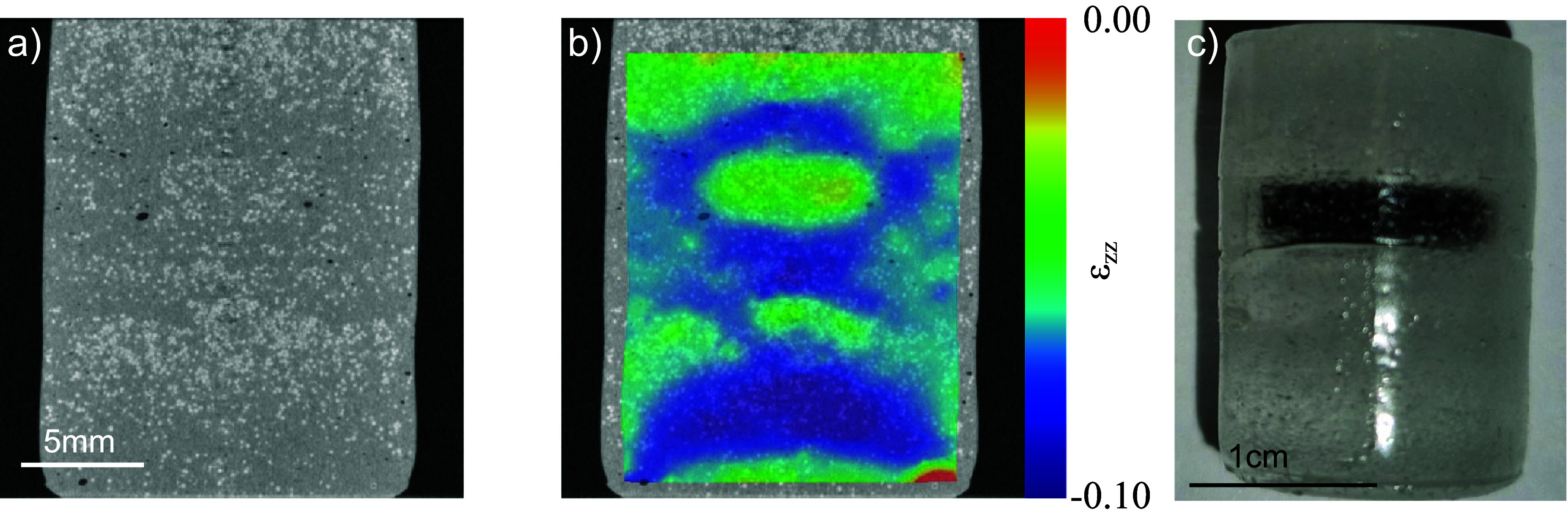
(a) Retrieved absorption CT slice through phantom B. PMMA microspheres are visible as white dots, while the inclusion is indistinguishable from the matrix. (b) *ϵ*_*zz*_ strain field after compressing phantom B by 1 mm. Although the inclusion is invisible in the absorption CT slice, the region of low strain in the upper half identifies the inclusion. (c) Photograph of phantom B. The inclusion was dyed with ink and is therefore visible in the photograph. This confirms the inclusion’s position in the strain field in (b).

Differences between the strain fields retrieved via FEA and those obtained from DVC with absorption contrast arise from several factors. These include the limited spatial resolution and precision of the DVC strain field, as well as the FEA approximation that assumes homogeneous Young’s moduli for both the matrix and the inclusion, whereas in reality, they vary depending on the local density of the PMMA spheres. Additionally, boundary conditions—such as the contact behaviour between the matrix and both the loading pistons and the inclusion—are not fully characterised. Nevertheless, these results demonstrate that, even in the presence of imperfect contact between the sample and the loading piston, the location and extent of a stiff inclusion can still be identified within the constraints of DVC strain precision and spatial resolution. A similar experiment was repeated using phantom B. Figure 6(a) shows a retrieved absorption CT slice through the phantom in the preloaded state. Although some clustering of the PMMA microspheres is evident, the absence of ${\mathrm{TiO}_2}$ powder in the inclusion prevents confident differentiation between the inclusion and the matrix using CT data alone. Figure 6(b) shows the retrieved *ϵ*_*zz*_ field after axially compressing the sample by 1.00 ± 0.04 mm. A region of low strain is visible in the middle top part of the image. The comparison of the CT slice to the photograph of the phantom shown in figure 6(c)—which shows the right-hand phantom from figure 1(a) and is included again here for the reader’s convenience to facilitate comparison with the strain field—confirms that this region is indeed the inclusion. It is striking that there are further regions of low strain in the very top and just below the middle of the strain field that are unexpected. These coincide with a higher density of PMMA microspheres, which are likely to have clumped together in the phantom making process, therefore locally increasing the stiffness of the material.

### 3.2. Strain precision

#### 3.2.1. Inclusion phantom.

**Figure 7.**
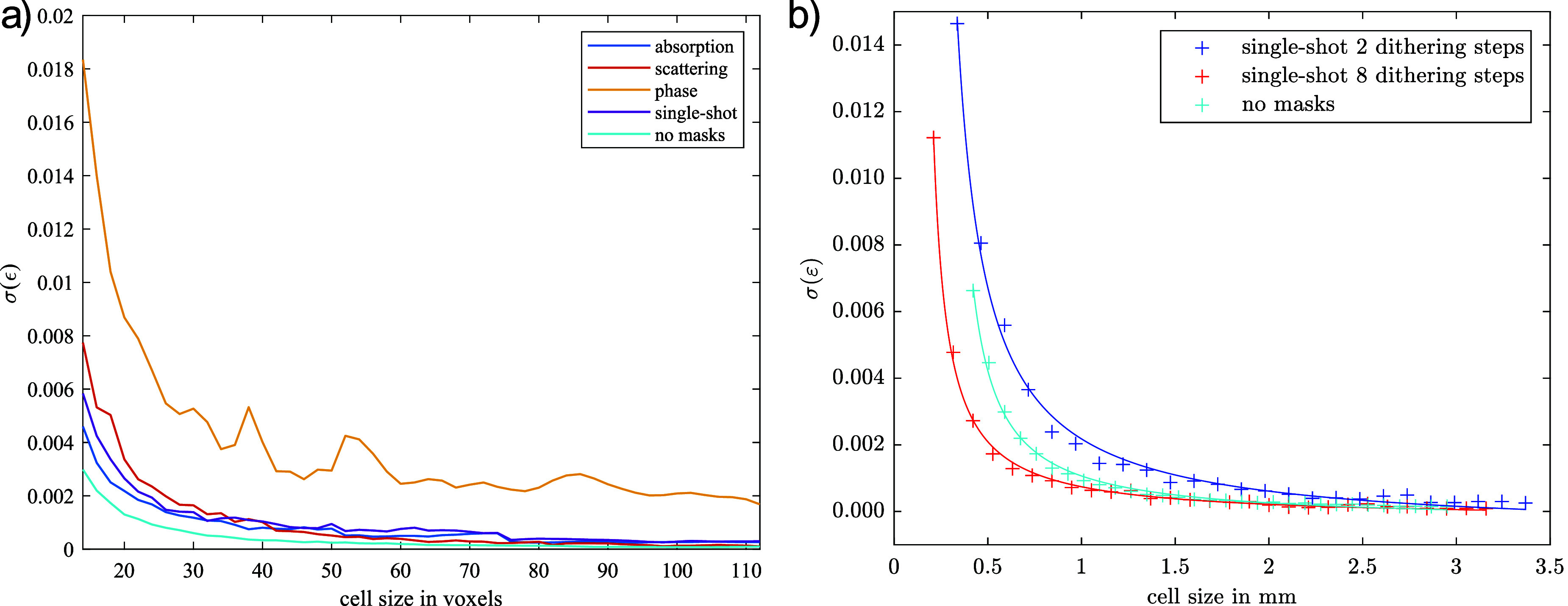
Plots of the mean of the standard deviations of the normal strains retrieved by DVC. (a) Comparison of the strain precision for different contrast mechanisms obtained from a five IC point and two dithering steps repeat scan of phantom A and a repeat scan removing the masks. The retrieved absorption reconstruction using no masks achieves the highest precision, whereas the artefacts in the retrieved phase reconstruction lead to a significantly decreased precision. (b) Comparison of the strain precision for different numbers of dithering steps. The curves represent the fit of the data to a reciprocal function of the form $y(x) = \frac{a_1}{x-a_2}+a_3$.

As demonstrated above, meaningful strain determination using phase CT reconstruction was not possible. This limitation is also reflected in the achievable strain precision. Figure 7(a) presents the DVC strain precision for the four different contrast mechanisms of EI, obtained experimentally through a zero-strain test on Phantom A. As expected, all four curves exhibit decreasing precision for finer mesh sizes. However, the precision in the retrieved phase reconstructions is two to three times lower due to strong streak artefacts (figure 4(a)).

Notably, the strain precision of the retrieved scattering reconstructions (figure 4(a)) appears comparable to that of the retrieved absorption reconstructions for coarse mesh sizes. This may be attributed to the inclusion remaining clearly visible, despite the significantly lower CNR for the interface between the matrix and background, which was 0.4 in the scattering reconstruction compared to 6.9 in the retrieved phase reconstruction. Additionally, the phantom’s borders and PMMA microspheres generate edge signals due to partial refraction of beamlets, leading to refraction-induced beamlet broadening. Consequently, the CNR for the interface between the PMMA spheres and matrix is 0.46, substantially higher than the corresponding CNR of 0.07 in the retrieved phase reconstruction.

As expected from visual inspection, absorption and single-shot reconstructions achieve the highest strain precision when using EI. For fine meshes, the retrieved absorption reconstruction appears to outperform the single-shot reconstruction, which may be attributed to the inherent low-pass filtering effect of single-shot phase retrieval or the improved photon statistics in the retrieved absorption reconstruction (five frames versus one). In line with observations from the compression experiment, strain precision is lowest for phase reconstructions. Moreover, this precision is likely overestimated, as artefacts in the zero-strain precision measurement may act as static features, artificially increasing the measured precision.

A repeat scan of phantom A was performed using the same number of projection angles (1000) as in the initial scan. Care was taken to obtain the same detector counts for one projection as the total detector counts for all five IC points of the EI scan, by using a 1 mm aluminium filter and adjusting the source current and exposure time. Because a column skipping mask was used in the EI setup, two dithering steps for the EI setup result in a sampling period equal to that of a scan without masks. Despite using the same photon statistics, not using masks seems to increase the strain precision even further. This could be due to fluctuations due to vibration-related mask movements, along with the need to combine two dithering steps that may suffer from minor inconsistencies from each other due to the above.

As mentioned earlier, EI allows the spatial sampling frequency to be increased at constant field of view by means of dithering. Figure 7(b) shows the influence of increasing the sampling frequency on the strain precision retrieved by DVC using the example of phantom A. To avoid aliasing effects, the reconstruction with eight dithering steps was performed using four times the number of projection angles compared to the reconstruction with two dithering steps. As already discussed, the strain precision is slightly higher without masks compared with performing two dithering steps in EI. However, increasing the spatial sampling frequency fourfold by using eight dithering steps increases precision, as the cells of the DVC mesh contain more voxels within the same physical size. The increased number of data points increases the precision of the correlation procedure. This allows for reducing the average cell size of the DVC mesh while maintaining an acceptable strain precision. Like this, the average cell size may be reduced until the Nyquist limit of the imaging system is reached, which in this case is a sampling period of half the aperture size, which corresponds to 16 dithering steps [38]. Eight dithering steps were selected to fully illuminate the sample, given the 1/8 ratio between the mask period and mask aperture, while also balancing acquisition time. Fitting the precision data to a reciprocal function of the form $y(x) = \frac{a_1}{x-a_2}+a_3$ enables the estimation of the minimum DVC cell size needed to achieve an acceptable strain precision of 0.002. Without masks, a cell size of at least 0.72 mm (17 voxels) is sufficient. For two dithering steps, a minimum cell size of 1.09 mm (26 voxels) is required, while for eight dithering steps, a cell size of at least 0.52 mm (49 voxels) is sufficient. Roughly a twofold increase in strain spatial resolution can be achieved when employing eight instead of two dithering steps. The reason for why the strain spatial resolution did not increase fourfold, in line with spatial sampling, could be related to the sample. The features in the phantoms result from the PMMA microspheres and the homogeneous gel wax/paraffin mixture filling the gaps between them. These features result in a particular spatial frequency spectrum being present in the CT slices, and as soon as the sampling frequency surpasses the maximum spatial frequency of the sample features, no extra information is gained from increased sampling.

#### 3.2.2. Biological tissue sample.

**Figure 8.**
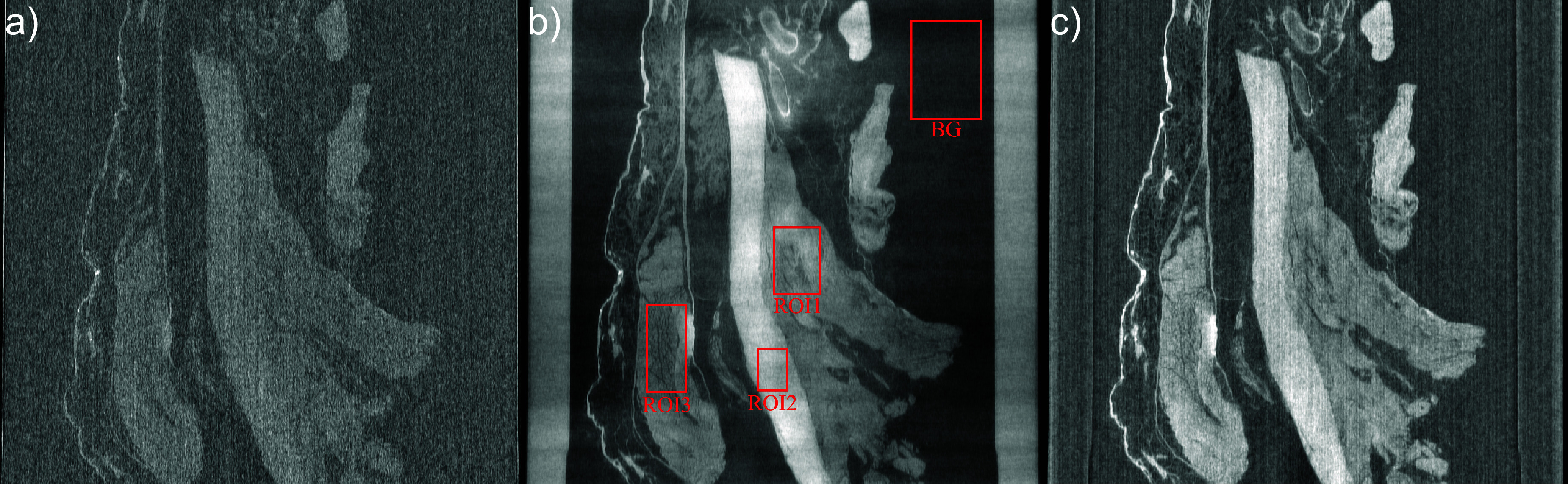
Longitudinal CT slices through chicken leg soft tissue fixed in 70% ethanol/water solution for different contrast mechanisms using eight dithering steps and three IC points. (a) Retrieved absorption. (b) Retrieved phase. (c) Single-shot retrieval. The red squares indicate the background (BG) and ROIs used for the calculation of the CNR for all images.

**Figure 9.**
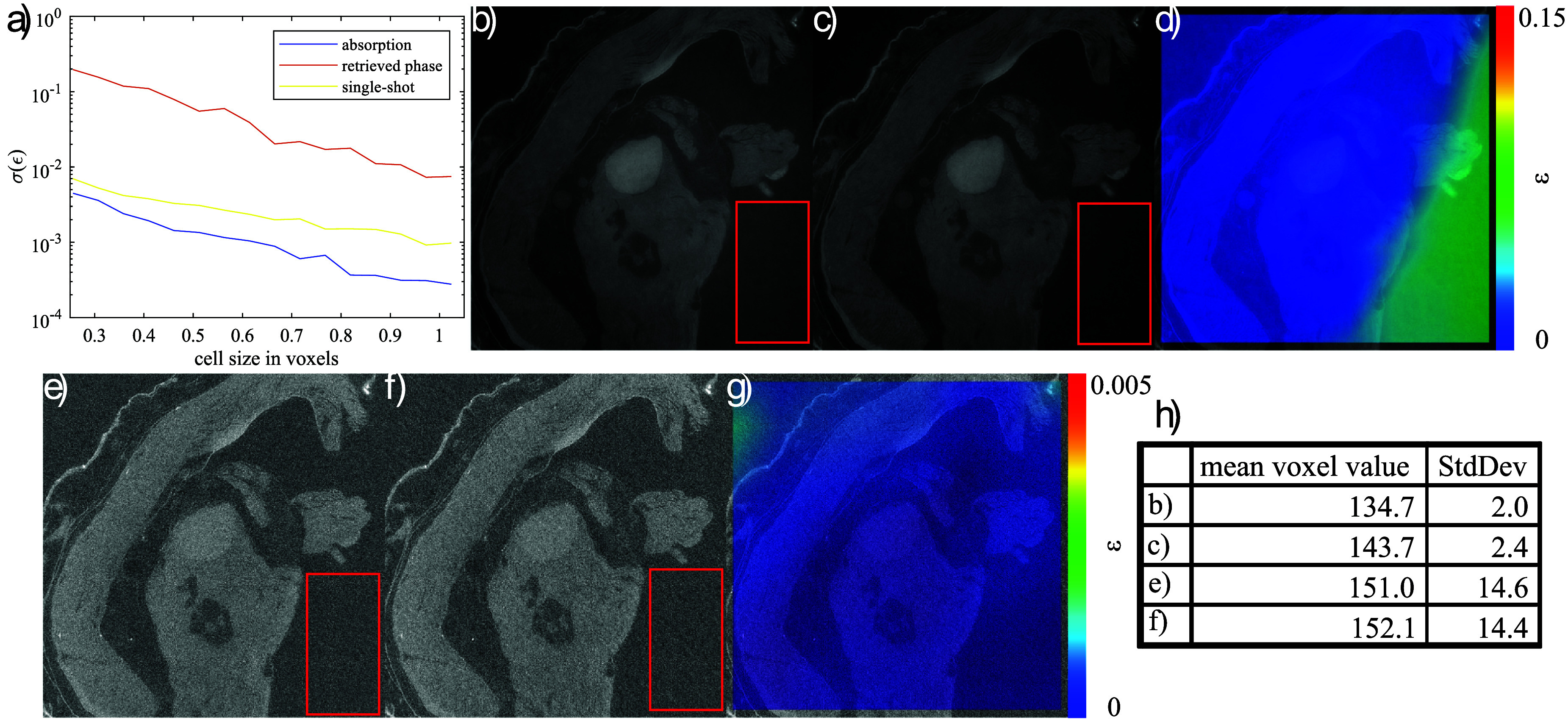
(a) Plots of the mean of the standard deviations of the retrieved normal strains for a zero strain test of the biological tissue sample. (b)–(c) Axial slices of a repeated retrieved phase CT reconstruction of the tissue sample. The red rectangle indicates a ROI in the background where the mean signal differs strongly between repeated retrieved phase reconstructions. (d) Magnitude of the normal strain vector retrieved from the phase repeat scans. (e)–(f) Axial slices of a repeated retrieved absorption CT reconstruction of the tissue sample. (g) Magnitude of the normal strain vector retrieved from the absorption repeat scans. (h) Table that compares the mean and standard deviation of the voxel values inside the red rectangle in (b)–(c), (e)–(f).

Contrary to our expectations, both phantoms exhibited good absorption contrast between the PMMA microspheres and the gel wax. To evaluate whether EI phase contrast could provide benefits for strain imaging in biological samples, where absorption contrast is typically expected to be poorer than for the phantoms, a scan was conducted on a tissue sample extracted from a chicken leg that was obtained from a commercial butcher. A compression test was not performed in this case, as the ground truth strain field would be unknown. The tissue sample was fixed in a 70% ethanol/water solution. The image volumes were reconstructed using 2000 projections, stitched from eight dithering steps and retrieved from three IC points. Furthermore, in order to smear out the streak artefacts seen in figure 4(b), vertical jittering was employed. This means that for each rotation angle the sample was moved vertically by a random, but known, multiple of the demagnified pixel size at the sample, which was then accounted for in the ASTRA reconstruction geometry. For this, a NEWPORT motor (XML210-S) with a positioning accuracy of 0.75 cm and bi-directional repeatability of 0.1 *µ*m was employed. The idea is that, similar to how horizontal jittering suppresses ring artefacts caused by non-linear pixels, vertical jittering could help to smear out artefacts caused by the absorbing bridges in the masks. Figure 8(a) shows a longitudinal retrieved absorption CT slice through the tissue sample. The image is noisy and it is hard to distinguish between different tissue types. In figure 8(b) the retrieved phase CT reconstruction of the same ROI is shown. The image is less noisy than the retrieved absorption CT and the different tissue types are easily distinguishable. Compared to the retrieved phase reconstruction in figure 4(b), the streak artefacts are now less pronounced because of the vertical jittering, but the image is still affected by parasitic low frequency brightness variations. While the retrieved phase reconstruction offers relatively high image quality and contrast, the single-shot reconstruction shown in figure 8(c) exhibits characteristics that are particularly well suited for DVC. Although the phase reconstruction displays higher overall visual quality—with certain features more easily distinguishable and higher CNR values than the other reconstructions, as reported in table 1—image quality in this study is assessed primarily in terms of its suitability for DVC. In this context, the single-shot image in figure 8(c) demonstrates relatively high CNR with clearly distinguishable tissue structures, and, most importantly, an absence of low-frequency artefacts, which are known to bias strain retrievals. This trade-off between conventional image quality and DVC-specific requirements is discussed further in relation to the results shown in figure 9.

**Table 1.** CNR for different ROIs (delineated in figure 8(b)) and contrast mechanisms.

CNR			
	Phase	Single-shot	Absorption
ROI1	11.8	5.4	1.2
ROI2	17.6	11.4	2.2
ROI3	6.4	6.9	1.5

Surprisingly, although the initial phase artefacts visible in figure 4(b) are strongly suppressed by the vertical jittering, performing a zero strain precision analysis with the chicken leg soft tissue sample leads to a similar result as with the inclusion phantom. Figure 9(a) shows that the strain precision using the retrieved phase reconstruction is more than an order of magnitude lower than for the absorption and single-shot reconstructions. In order to understand this, the differences between repeat scans with different contrasts were analysed in more detail. In figures 9(b)–(c) and (e)–(f) corresponding axial slices from the retrieved phase and absorption reconstructions are shown. Similar to the longitudinal view, a low-frequency brightness variation is visible in the retrieved phase slices of both repeat scans. However, its appearance differs between the two scans and is absent in the corresponding retrieved absorption slices. The voxel statistics inside the ROI, indicated by the red rectangle, were calculated (figure 9(h)). Notably, in the retrieved phase reconstructions, the ratio between the standard deviation and the difference in the mean voxel values is only 0.24, whereas in the retrieved absorption reconstructions, this ratio is 13.18. Using DVC, this strong signal variation in the retrieved phase reconstruction due to the low-frequency brightness variations competes with the signal due to sample features, and therefore leads to an apparent deformation being calculated in the zero strain test. The measured strain distribution of the zero strain test using phase in figure 9(d) supports this claim because the retrieved strain, which should ideally be zero everywhere, is highest in regions where no sample is present—that is, where the low-frequency artefact dominates the CT image. This demonstrates that although the CNR of the image details is higher for the retrieved phase images, the DVC strain precision is lower because it is dominated by the low-frequency artefact caused by the phase integration incorrectly measured strain is highest in the regions where sample features are absent. When comparing this strain distribution to the absorption zero strain test in figure 9(g), it is notable that the scale of the measured strains is one order of magnitude higher. Furthermore, in the absorption case the incorrectly measured strain is much more equally distributed and seems to originate from the underlying noise and not from an imaging artefact. In summary, the worse performance of phase in the accuracy of strain measurements via DVC can be attributed to the spatially random appearance of phase integration artefacts.

## 4. Conclusion

We demonstrated, using EI absorption contrast, that an otherwise invisible inclusion could be identified through strain contrast in phantoms with tissue-realistic Young’s moduli. By performing FEA and quantifying the precision in strain retrieved via DVC, we confirmed that the strain contrast between mechanically realistic tumour and healthy tissue is sufficiently high to allow for a clear distinction between healthy and tumour tissue, provided that features are visible in absorption contrast.

The potential of EI phase contrast imaging to improve strain retrieval precision was investigated. For both relatively strongly absorbing inclusion phantoms and a biological tissue sample, strain uncertainties were significantly higher when using retrieved phase reconstructions compared to retrieved absorption reconstructions. In a compression test of the phantom, the inclusion could not be identified. This outcome is attributed to strong artefacts in the retrieved phase reconstructions. Beyond the impact of bridges and other suboptimal features in the mask fabrication, phase integration artefacts played a key role. The random nature of these artefacts results in uncorrelated positions across repeated scans. To fully leverage the higher CNR provided by EI phase contrast, further research is needed to eliminate artefacts caused by the EI masks and to regularise phase integration, thereby reducing background variations that can confuse the image correlation algorithm.

Despite these challenges, it was demonstrated that increasing the spatial sampling rate through additional dithering steps enables the use of finer DVC meshes, achieving higher strain spatial resolution at a given precision level compared to conventional absorption CT without masks. However, acquiring a fully dithered single IC point CT scan (in this setup, 16 dithering steps to satisfy the Nyquist sampling theorem) remains time-consuming (∼24 h). Future investigations should focus on reducing scan time through techniques such as fly scans, shorter exposure times, and undersampling schemes like cycloidal CT [39], to assess their impact on strain retrieval precision.

## Data Availability

The data that support the findings of this study are openly available at the following URL/DOI: https://doi.org/10.5522/04/28554059.v1.
